# Chlorinative stress in age-related diseases: a literature review

**DOI:** 10.1186/s12979-017-0104-5

**Published:** 2017-11-14

**Authors:** Marco Casciaro, Eleonora Di Salvo, Elisabetta Pace, Elvira Ventura-Spagnolo, Michele Navarra, Sebastiano Gangemi

**Affiliations:** 1 School and Division of Allergy and Clinical Immunology, Department of Clinical and Experimental Medicine, Azienda Ospedaliera Universitaria Policlinico “G. Martino”, University of Messina, Messina, Italy; 2 IBIM-CNR Institute of Biomedicine and Molecular Immunology, National Research Council, 90100 Palermo, Italy; 3 Legal Medicine Section, Department for Health Promotion and Mother-Child Care, University of Palermo, Via del Vespro, 129, 90127 Palermo, Italy; 4 Department of Chemical, Biological, Pharmaceutical and Environmental Sciences, University of Messina, Messina, Italy

**Keywords:** Oxidative stress, Myeloperoxidase, Chlorine, Chlorinative stress, Chlorination, Aging, Age, Protein damage, Hypochlorous acid, Inflammation

## Abstract

Aging is an agglomerate of biological long-lasting processes that result being inevitable. Main actors in this scenario are both long-term inflammation and oxidative stress. It has been proved that oxidative stress induce alteration in proteins and this fact itself is critically important in the pathophysiological mechanisms leading to diseases typical of aging. Among reactive species, chlorine ones such as hypochlorous acid (HOCl) are cytotoxic oxidants produced by activated neutrophils during chronic inflammation processes. HOCl can also cause damages by reacting with biological molecules. HOCl is generated by myeloperoxidase (MPO) and augmented serum levels of MPO have been described in acute and chronic inflammatory conditions in cardiovascular patients and has been implicated in many inflammatory diseases such as atherosclerosis, neurodegenerative conditions, and some cancers. Due to these data, we decided to conduct an up-to-date review evaluating chlorinative stress effects on every age-related disease linked; potential anti-oxidant countermeasures were also assessed. Results obtained associated HOCl generation to the aging processes and confirmed its connection with diseases like neurodegenerative and cardiovascular pathologies, atherosclerosis and cancer; chlorination was mainly linked to diseases where molecular (protein) alteration constitute the major suspected cause: i.e. inflammation, tissue lesions, DNA damages, apoptosis and oxidative stress itself. According data collected, a healthy lifestyle together with some dietary suggestion and/or the administration of nutracetical antioxidant integrators could balance the effects of chlorinative stress and, in some cases, slow down or prevent the onset of age-releated diseases.

## Background

Aging is an agglomerate of biological long-lasting processes that result being inevitable. It is correlated to gradual and autonomous biochemical and physiological changes, which often leads to an increased diseases susceptibility. The key players in this scenario are both long-term inflammation and oxidative stress [1]. The aging process is dynamic and characterized by a continuous remodelling. DNA repair, apoptosis, immune response, oxidative stress and inflammation contribute to this dynamic process [2]. The natural deduction is that oxidative stress and aging are strictly connected.

Reactive oxygen species (ROS) are a critical class of DNA detrimental agents and, unfortunately, they are constantly produced in human cells in response to toxicant either generated from our own metabolism and/or exposure to environmental agents [3–5]. ROS include different chemical species such as superoxide anion radical (O2-), hydrogen peroxide (H2O2), hydroxyl radical (·OH), and singlet oxygen (O2). Mitochondrion and NADPH oxidases are contemplated major sources of ROS generation in cells. In mitochondria, electrons slipping from the electron transport chain, in the course of mitochondrial respiration, can combine with oxygen in generating O2; O2 itself could subsequently be converted to H2O2 by superoxide dismutase (SOD) [6]. The oxidative process could affect many redox-sensitive biological molecules (i.e. amino acids) depending on the site of ROS production [5, 6]. Inflammatory cells, recruited after a chemical, physical or biological damage, promote the activation or the induction of different oxidant-generating enzymes. These enzymes generate high levels of reactive oxygen, nitrogen, and halogen species on site of the inflammation process. Some of these species are i.e. superoxide anion, nitric oxide, peroxynitrite, hydrogen peroxide, hypochlorous acid, and hypobromous acid. Their main objective is neutralizing invading pathogens, but often their action could lead to the DNA damage of host cells as side effect [5, 6]. It has been proved that oxidative stress induce alteration in proteins and this fact itself is critically important in the pathophysiological mechanisms leading to diseases typical of aging, extending from atherosclerosis to neurodegenerative disorders [7] as well as other inflammatory and immunological diseases [8, 9].

The maintenance of optimal conditions in an organism in order to contrast aging is accomplished by a complex network of longevity assurance processes that are controlled by Vitagenes, a group of genes aimed at preserving cellular homeostasis during stressful conditions; in particular Vitagenes encode the genes for the formation of Hsp (heat shock proteins), useful to counteract the formation of miticondrial ROS and thus, the progression of unsuccessful aging [10]. Vitagens have also been studied in diseases typical of the old age such as neurodegenerative ones [11]. In recent years, many scientific researches were aimed to refine new techniques based on proteomics, which lead to the discovery of new proteins and molecules having a key role in oxidative stress and aging processes, and for the identification of selected proteins to be used in a specific therapeutic targets [12].

Among the above cited reactive species, chlorine ones such as hypochlorous acid (HOCl) are cytotoxic oxidants produced by activated neutrophils during chronic inflammation processes [13]. Neutrophils and some monocyte-macrophage line cells generate HOCl by the myeloperoxidase (MPO) enzyme intervention. HOCl is a powerful cytotoxic oxidant acting a main role in fighting microbial pathogens. By the way it can also cause damages by reacting with biological molecules (i.e. amino acids, lipids, nucleic acids) sustaining its inflammation. Proteins are the most common targets of HOCl and for this reason changes induced in their sub-elements (peptides and amino acids) have been largely examined [5, 6, 13]. HOCl oxidizes cysteine and methionine, leading to the production of disulfides, oxyacids, sulfoxides, and molecules where sulfur is linked to nitrogen. Other reactive species could generate oxygenated sulfur products too, but they are useless as biomarkers for HOCl-induced damage [14].

HOCl is generated by the heme enzyme myeloperoxidase [15]. MPO is a glycosylated heme-enzyme stored in neutrophils and macrophages azurophilic granules; these granules have a powerful bactericidal action which is mediated by the production of hypochlorous acid from hydrogen peroxide and chloride ions. MPO could be secreted in the extracellular space; augmented serum levels of MPO have been described in acute and chronic inflammatory conditions in cardiovascular patients and have been implicated in many inflammatory diseases such as atherosclerosis, neurodegenerative conditions, and some cancers [16–20]. Due to these data, we decided to search literature trying to delineate a complete and up to date overview about hypochlorous acid and chlorinative stress effects in aging and in all the age related diseases linked. Moreover, potential anti-oxidant countermeasures were also assessed.

## Methods

This literature review was conducted employing MEDLINE database. On this database, we searched for articles from inception to march 2017 using key terms related to aging and chlorinative stress.

It was decided to read the abstract of articles whose titles indicated that they might have examined HOCl involvement and the role of chlorinative stress in age-related diseases. The entire article was read if the abstract indicated that the article potentially met the inclusion criteria. Lastly, we reviewed and searched references of the selected articles. Articles were included in the present review according to the following inclusion criteria: English language, publication in peer reviewed journals, research paper. Articles were excluded by title, abstract or full text for irrelevance to the topic in question. Two authors (MC, EDS) performed the initial search and independently reviewed and selected the references based on the inclusion and exclusion criteria. Principal outcomes of interest included original studies concerning HOCl involvement and/or chlorinative stress in age-related diseases.

### Chlorinative stress and cardiovascular diseases

Due to well-known HOCl generation by myeloperoxidase, Daugherty et al. in 1994. studied atherosclerotic lesions where they detected the enzyme MPO; their data sustained that MPO induced LDL oxidation by pathways involving HOCl and promoting atherogenesis [21]. These data were studied thoroughly by Hazen et al. which demonstrated that HOCl produced by the system myeloperoxidase-H2O2-Cl oxidated LDL l-tyrosine. Oxidated LDL were known to have a main role in converting macrophages into foam cells and in forming atherosclerotic lesions typical of aged people [22]. In a successive paper, they also demonstrated that 3-chlorotyrosine was a marker of chlorination at sites of inflammation [7]. Also the eosinophil peroxidase (EPO) was thought being implicated in provoking oxidative tissue damage in many conditions (i.e. asthma, allergic inflammatory disorders, cancer, infections). But Wu et al. reported that EPO generated reactive nitrogen species by direct oxidation of NO 2 and not by secondary oxidation of NO 2 by HOCl [23]. Every paper reported seemed to address the chlorinating stress as responsible of cardiovascular-age related damage so next step was demonstrating if oxidative stress HOCl-related could induce endothelial dysfunction by interfering with the NO synthetic pathway. Zhang et al. noticed that a treatment with HOCl caused the inhibition of aortic relaxation correlated to HOCl concentration. In their experiments, they also dosed endothelial NO synthase (NOS III) after the administration of HOCl then supplemented rats with L-arginine and reported a complete inversion of HOCl inhibitory effect vessel relaxation [24]. As Oxidized lipoproteins act a main role in atherosclerosis and high levels of 3-chlorotyrosine were demonstrated being in atherosclerotic lesion, Bergt et al. studied how activated phagocytes chlorinated specific region in HDL by mass spectrometry. Results obtained demonstrated how HOCl selectively targeted tyrosine residues nearby primary amino-groups in proteins. This oxidation process performed by phagocytes lead to damage host tissue during inflammatory diseases (i.e. atherosclerosis) [14]. As demonstrated by Cook et al., SERCA activity is fundamental in human homeostasis; it is blocked by oxidant agents and deregulated in aged tissues and cardiovascular pathologies. They reported that HOCl targeted thiols and provoked cellular impairment. They speculated that HOCl could inhibit SERCA activity by thiol oxidation and generated cytosolic Ca2+ augmented levels in artery endothelial cells [17]. Ismael et al. shown that exposure of macrophages to HOCl and HOSCN or to LDL already modified by these chlorinative stress agents caused a compromised lysosomal enzyme function reducing both proteolytic capacity and decrease cholesteryl ester hydrolysis; according the authors these events could conduct to the accumulation of protein and lipids in the arterial wall fundamental for the development of atherosclerosis [25].

Therefore, Wang et al. in vivo studies demonstrated that damaged LV-tissue by the induction of an acute myocardial infarction lead to the recruitment and activation of neutrophils and concomitant increase in MPO-activity and consequently of the HOCl; the 3-Cl-Tyr formed by the HOCl modification of the heme protein Mb impaired the protein’s affinity for binding oxygen in the myocardium [26].

From other studies emerged that MPO augmented activity during inflammatory processes could worsen diseases such as atherosclerosis and reperfusion injury; in fact, an increased release of hypochlorite contributed to the damage observed in these pathologies [24, 27–30]. On these basis Sand et al. evaluated the effects of hypochlorite and H2O2 on 1-adrenoceptor, ET-1 receptors and M2 receptors processes on mice. They concluded that formation of hypochlorite provoked the amplification of the oxidative capabilities of H2O2 enhancing the damage of endothelial physiology. Hypochlorite also interfered with the normal transduction of the 1-adrenoceptor by altering the coupling of the muscarinic M2 receptor to the G-proteins favouring the progression of inflammation-associated pathologies in the cardiovascular system [31].

### Chlorinative stress and other aging conditions

On the other hand it was also important understanding the inflammatory mechanisms HOCl - associated, so Raftery et al. demonstrated that HOCl produced by myeloperoxidase and H2O2 and by phorbol 12-myristate 13-acetate stimulated and activated neutrophils. HOCl oxidation during inflammation caused the formation of sulfamide monomers having chemotactic activity for neutrophils in inflammation. These changes in human proteins could, according Raftery et al. potentially play a critical role in physiological and pathological processes fundamental in aging and age-related diseases [32]. In a rat hepatocytes experiment, Mallis et al. reported that HOCl generated in a non-reversible way oxidized forms of carbonic anhydrase III in case of low doses or absence of glutathione (GSH). As it is known that there is a physiological reduction of glutathione in aged animals, these condition may together with other mechanisms favourite a higher irreversible protein oxidation [33]. Strosovà et al. 2005., studied the effects of HOCl in rabbit skeletal sarcoplasmic reticulum (SR) speculating an aging model. It was found that HOCl blocked Ca^2+^-ATPase activity. It also oxidated SH groups and formed protein carbonyls. On the other side they tested and demonstrated the antioxidant effect of stobadine (at least as much as lipoic acid), trolox and Pycnogenol [34]. Hazell et al., wanted to determine if HOCl could be involved in protein modifications associated to age-related eye disease such as nuclear cataract. In the human lens samples analysed no chlorotyrosine derivates could be detected and no myeloperoxidase activity trace could be found either [35].

In the attempt to report the role of neutrophils as source of oxidative stress in rheumatoid arthritis (RA), Baskol et al. 2006., described how advanced oxidation protein products (AOPP) are formed by the interaction of HOCl/HOCl and proteins. In the samples analysed the protein oxidative damage corresponded to increased levels of AOPP and nominated them as marker of oxidative stress. According their data, neutrophils, which produce great quantities of chlorinated oxidants by MPO, could augment serum AOPP levels and play a fundamental role in the pathogenesis of RA by generating pro-inflammatory mediators [36]. Although its known induction of oxidant intermediates Leung et Al. decided to study skin and NF-kB effects of topical application of HOCl. They reported HOCl capacity to block NF-kB signalling and to attenuate NF-kB related disease as acute radiation dermatitis and skin aging process [37].As regards the young and the elderly’s immune system, many years ago it emerged that neutrophil extracellular traps (NETs) are part of the defensive mechanism of neutrophils; their formation requires reactive oxygen species presence. Hazeldine et al. demonstrated that NET generation in response to HOCl was lower only in aged patients. A deficit on NET formation could contribute to a higher incidence of infection in older adults [38]. Superoxide (O2-) and hydrogen peroxide (H2O2) are consequences of hyperglycemia; during last years was clarified their role in the apoptosis of endothelial cells and in causing diabetic vascular injury. This damage is the result of endothelial dysfunction and vascular complications. As NADPH oxidase-derived ROS and vascular-bound MPO are increased in diabetic vessels, MPO/NADPH oxidase/H2O2/HOCl could constitute a main path in diabetic vascular damages. According Tian et al. blocking the MPO-NADPH oxidase-HOCl pathway could be a novel therapeutic strategy for the prevention and the recovery of vascular diseases [39].

MPO and its consequent product HOCl resulted involved in the pathophysiology of neurodegenerative diseases. In fact, MPO is expressed with increased levels in the cerebral tissue of patients affected by Alzheimer disease (AD) and this enzyme appears being catalytically active [40]. Not present in normal brains of old patients, MPO was detectable in amyloid plaques of AD patients. It was speculated a model where MPO expression in astrocytes promoted a HOCl related damage, contributing to neuronal damage and cognitive impairment [40, 41].

## Discussion

It has always been known that oxidation of proteins was a detrimental process leading to the damage of human tissues. Both inflammation and the consequent cellular damage are two of the most threatening causes of the unsuccessful aging process. Oxidative stress has always been designated as an inflammatory source leading to many aging diseases and the stress caused by hypochlorous acid is a consistent part of it. Since 1991 with the first studies involving MPO, it appeared to be quite clear the importance of one of myeloperoxidase “normal” products, the HOCl, in the above cited oxidation process. The word “chlorination” was used to describe the modification induced in molecules by HOCl intervention; “chlorinative stress” expression was chosen to define the sum of HOCl pathological interaction in a physiological organism [14, 21, 42, 43]. Several of the diseases induced by this stress either favourite or provoke age-related diseases. The generation of HOCl was demonstrated to interfere with lipoproteins with a consequent oxidation resulting in the conversion of macrophages in foam cells and in the acceleration of the atherosclerotic process [7, 14, 21, 22]. HOCl also induced endothelial dysfunction by interfering with the NO synthetic pathway. This event blocked vessel relaxation and together with the pro-atherosclerotic effect above described could generate the basis for many heart diseases [23, 24]. Chlorination could also augment inflammation at damage sites by recruiting neutrophils and induce muscle-associated pathologies by irreversibly blocking carbonic anhydrase III, creating so a vicious circle involving also calcium balance and smooth muscle contraction [17, 32–34]. MPO-dependent chlorinating stress it was demonstrated to be also related to the presence of ANCA antibodies; in fact, MPO can be individuated by MPO-ANCA, and in consequence neutrophils burst and degranulation are enhanced with a subsequent HOCl MPO-dependent generation [44–47]. It also emerged that neutrophils recruitment and consequent HOCl production in myocardium after IMA could modify heme proteins and compromise the post-infarction recovery contributing to the progression of heart failure [26]. By literature analysis other than heart-related diseases, HOCl seemed also to favourite the production of pro-inflammatory mediators in AR by forming AOPP agents [36]. Recently hypochlorous acid emerged to be not so effective in older people in generating neutrophils inflammatory response as in younger ones [38].

Although HOCl was shown being detrimental, studies conducted during last years exonerated it from being a cataract causing-agent and from generating reactive nitrogen species via EPO [23, 35]. HOCl topic application could even prevent skin aging [37]. Novel data supported outdated results and confirmed the hypothesis of a serious endothelial damage induced by chlorinative stress; above all, authors deepened the detrimental effects induced by hyperglycaemia and by neutrophils intervention immediately after an acute myocardial injury [26, 39]. During last years, it emerged also the role of hypoclorous acid produced by MPO in causing neuronal apoptosis and damages [40, 41].

**Fig. 1:**
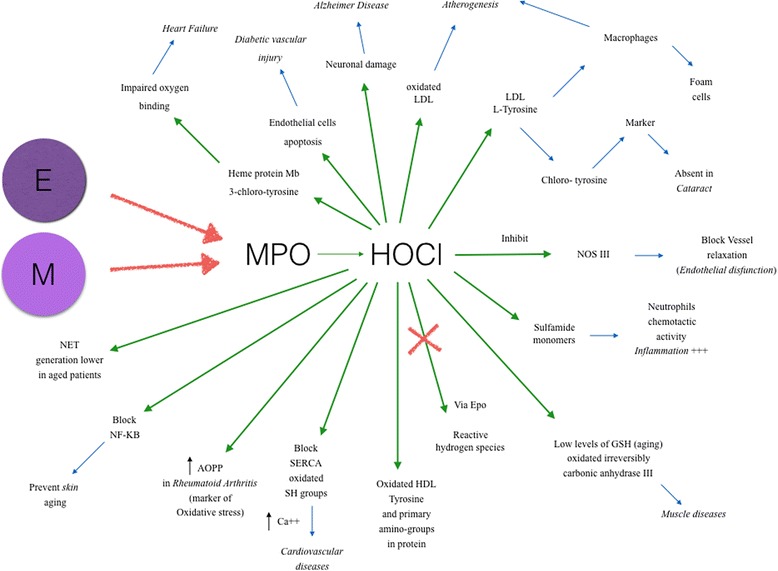
Effects of hypochlorous acid in age-related diseases

These findings associated HOCl generation to aging processes and confirmed its connection with some diseases such as neurodegenerative and cardiovascular pathologies, atherosclerosis and cancer (Fig. 1); chlorination was mainly linked to diseases where molecular alterations constituted the major suspected cause: i.e. inflammation, tissue lesions, DNA damages, apoptosis and oxidative stress itself [17].

Novel, preventive and, in some cases, therapeutic approaches should be planned in order to contrast aging effects and the development of the above cited diseases. A specific low chlorine diet, rich in natural and endogenous antioxidant foods could favourite this prevention [48]. For example, Taurine was demonstrated to be a physiological primary scavenger of HOCl; since serum and urine taurine levels in elderly patients with chronic inflammatory disorders were found to be reduced, an adequate level of the amino acid inside the body may be useful in order to prevent age-related diseases [49].

Another innovative therapeutic approach could be provided by the administration natural antioxidants such as Resveratrol, Green Tea, Curcumin and Ferulic Acid; their intervention could be protective versus several diseases capable of causing tissue damage and the generation of free radical (i.e. neurodegeneration) [50].

Recent advances in the nutraceutical field highlighted the importance of some dietary integrator in aging-related pathologies. Some of these integrators such as ascorbic acid, docosahexaenoic acid (DHA) and in general low molecular weight antioxidant, associated with an adequate diet and sport could efficiently contrast ROS formation [51].

One of the most interesting molecules observed in the studies was 3-chlorotyrosine and according us should be studied in depth. Using 3-chlorotyrosine as serum and urine chlorination marker could be useful in monitoring neutrophils and macrophages activity (as major MPO sources) in some pathological phases; clarifying their involvement and diminishing their activity could finally lead to the down-regulation of HOCl production.

## Conclusion

A healthy lifestyle together with some dietary suggestion and/or the administration of nutracetical antioxidant integrators could balance the effects of chlorinative stress and, in some cases, slow down or prevent the onset of age-releated diseases.

Once understood that ROS formation is in part responsible for cell damages and senescence, the next step will be focusing on studies of neurogenetics, proteomic and concerning the identification of new biomarkers. In order to contrast the most severe diseases, probably, the development of more specific ligands targeting the molecules involved in oxidative stress should have the priority. In accordance of what described above, one of these eligible targets could be the leading actor of this review, the product of MPO: hypoclorous acid [52].

## References

[1] Sharma S, Ebadi M (2014). Significance of metallothioneins in aging brain. Neurochem Int.

[2] Minciullo PL (2016). Inflammaging and anti-Inflammaging: the role of cytokines in extreme longevity. Arch Immunol Ther Exp.

[3] D'Autreaux B, Toledano MB (2007). ROS as signalling molecules: mechanisms that generate specificity in ROS homeostasis. Nat Rev Mol Cell Biol.

[4] Krause KH (2007). Aging: a revisited theory based on free radicals generated by NOX family NADPH oxidases. Exp Gerontol.

[5] Brieger K (2012). Reactive oxygen species: from health to disease. Swiss Med Wkly.

[6] Yu Y (2016). Occurrence, biological consequences, and human health relevance of oxidative stress-induced DNA damage. Chem Res Toxicol.

[7] Hazen SL (1997). Mass spectrometric quantification of 3-chlorotyrosine in human tissues with attomole sensitivity: a sensitive and specific marker for myeloperoxidase-catalyzed chlorination at sites of inflammation. Free Radic Biol Med.

[8] Di Lorenzo G (2013). Differences in the behavior of advanced glycation end products and advanced oxidation protein products in patients with allergic rhinitis. J Investig Allergol Clin Immunol.

[9] Gangemi S (2015). Oxidative stress markers are increased in patients with mastocytosis. Allergy.

[10] Calabrese V (2012). Cellular stress responses, hormetic phytochemicals and vitagenes in aging and longevity. Biochim Biophys Acta.

[11] Calabrese V (2016). Major pathogenic mechanisms in vascular dementia: roles of cellular stress response and hormesis in neuroprotection. J Neurosci Res.

[12] Calabrese V (2015). Analytical approaches to the diagnosis and treatment of aging and aging-related disease: redox status and proteomics. Free Radic Res.

[13] Whiteman M (2008). Do mitochondriotropic antioxidants prevent chlorinative stress-induced mitochondrial and cellular injury?. Antioxid Redox Signal.

[14] Bergt C (2004). Lysine residues direct the chlorination of tyrosines in YXXK motifs of apolipoprotein A-I when hypochlorous acid oxidizes high density lipoprotein. J Biol Chem.

[15] Marcinkiewicz J (2000). Antimicrobial and cytotoxic activity of hypochlorous acid: interactions with taurine and nitrite. Inflamm Res.

[16] Sharma RN, Goel S (2007). Chlorinated drinking water, cancers and adverse health outcomes in Gangtok, Sikkim, India. J Environ Sci Eng.

[17] Cook NL (2012). Myeloperoxidase-derived oxidants inhibit sarco/endoplasmic reticulum Ca2+−ATPase activity and perturb Ca2+ homeostasis in human coronary artery endothelial cells. Free Radic Biol Med.

[18] Straface E (2012). Does oxidative stress play a critical role in cardiovascular complications of Kawasaki disease?. Antioxid Redox Signal.

[19] Cabassi A (2015). Myeloperoxidase-related chlorination activity is positively associated with circulating Ceruloplasmin in chronic heart failure patients: relationship with Neurohormonal, inflammatory, and nutritional parameters. Biomed Res Int.

[20] Ray RS, Katyal A (2016). Myeloperoxidase: bridging the gap in neurodegeneration. Neurosci Biobehav Rev.

[21] Daugherty A (1994). Myeloperoxidase, a catalyst for lipoprotein oxidation, is expressed in human atherosclerotic lesions. J Clin Invest.

[22] Hazen SL, Heinecke JW (1997). 3-Chlorotyrosine, a specific marker of myeloperoxidase-catalyzed oxidation, is markedly elevated in low density lipoprotein isolated from human atherosclerotic intima. J Clin Invest.

[23] Wu W, Chen Y, Hazen SL (1999). Eosinophil peroxidase nitrates protein tyrosyl residues. Implications for oxidative damage by nitrating intermediates in eosinophilic inflammatory disorders. J Biol Chem.

[24] Zhang C (2001). Endothelial dysfunction is induced by proinflammatory oxidant hypochlorous acid. Am J Physiol Heart Circ Physiol.

[25] Ismael FO (2016). Role of Myeloperoxidase oxidants in the modulation of cellular Lysosomal enzyme function: a contributing factor to macrophage dysfunction in atherosclerosis?. PLoS One.

[26] Wang XS (2016). Neutrophils recruited to the myocardium after acute experimental myocardial infarct generate hypochlorous acid that oxidizes cardiac myoglobin. Arch Biochem Biophys.

[27] Okabe E (1993). The effect of hypochlorous acid and hydrogen peroxide on coronary flow and arrhythmogenesis in myocardial ischemia and reperfusion. Eur J Pharmacol.

[28] Raschke P (1993). Postischemic dysfunction of the heart induced by small numbers of neutrophils via formation of hypochlorous acid. Basic Res Cardiol.

[29] Mian KB, Martin W (1997). Hydrogen peroxide-induced impairment of reactivity in rat isolated aorta: potentiation by 3-amino-1,2,4-triazole. Br J Pharmacol.

[30] Jaimes EA, Sweeney C, Raij L (2001). Effects of the reactive oxygen species hydrogen peroxide and hypochlorite on endothelial nitric oxide production. Hypertension.

[31] Sand C (2003). Effects of hypochlorite and hydrogen peroxide on cardiac autonomic receptors and vascular endothelial function. Clin Exp Pharmacol Physiol.

[32] Raftery MJ (2001). Novel intra- and inter-molecular sulfinamide bonds in S100A8 produced by hypochlorite oxidation. J Biol Chem.

[33] Mallis RJ (2002). Irreversible thiol oxidation in carbonic anhydrase III: protection by S-glutathiolation and detection in aging rats. Biol Chem.

[34] Strosova M, Skuciova M, Horakova L (2005). Oxidative damage to Ca2+−ATPase sarcoplasmic reticulum by HOCl and protective effect of some antioxidants. Biofactors.

[35] Hazell LJ (2002). Is hypochlorous acid (HOCl) involved in age-related nuclear cataract?. Clin Exp Optom.

[36] Baskol G (2006). Investigation of protein oxidation and lipid peroxidation in patients with rheumatoid arthritis. Cell Biochem Funct.

[37] Leung TH (2013). Topical hypochlorite ameliorates NF-kappaB-mediated skin diseases in mice. J Clin Invest.

[38] Hazeldine J (2014). Impaired neutrophil extracellular trap formation: a novel defect in the innate immune system of aged individuals. Aging Cell.

[39] Tian R (2017). Myeloperoxidase amplified high glucose-induced endothelial dysfunction in vasculature: role of NADPH oxidase and hypochlorous acid. Biochem Biophys Res Commun.

[40] Lu N (2016). Inhibition of myeloperoxidase-mediated oxidative damage by nitrite in SH-SY5Y cells: relevance to neuroprotection in neurodegenerative diseases. Eur J Pharmacol.

[41] Maki RA (2009). Aberrant expression of myeloperoxidase in astrocytes promotes phospholipid oxidation and memory deficits in a mouse model of Alzheimer disease. J Biol Chem.

[42] Marcinkiewicz J (1991). Enhancement of immunogenic properties of ovalbumin as a result of its chlorination. Int J BioChemiPhysics.

[43] Guo Y, Schneider LA, Wangensteen OD (1995). HOCl effects on tracheal epithelium: conductance and permeability measurements. J Appl Physiol (1985).

[44] Foote CS, Goyne TE, Lehrer RI (1983). Assessment of chlorination by human neutrophils. Nature.

[45] Falk RJ (1990). Anti-neutrophil cytoplasmic autoantibodies induce neutrophils to degranulate and produce oxygen radicals in vitro. Proc Natl Acad Sci U S A.

[46] Xu PC (2012). Influence of myeloperoxidase-catalyzing reaction on the binding between myeloperoxidase and anti-myeloperoxidase antibodies. Hum Immunol.

[47] Wang J, et al. Deglycosylation influences the oxidation activity and antigenicity of myeloperoxidase. Nephrology (Carlton). 2016. doi:10.1111/nep.12926. 10.1111/nep.12926 27643667

[48] Arrigo T (2015). Role of the diet as a link between oxidative stress and liver diseases. World J Gastroenterol.

[49] Yamori Y (2004). Fish and lifestyle-related disease prevention: experimental and epidemiological evidence for anti-atherogenic potential of taurine. Clin Exp Pharmacol Physiol.

[50] Calabrese V (2010). The hormetic role of dietary antioxidants in free radical-related diseases. Curr Pharm Des.

[51] Capó X (2016). Effects of almond- and olive oil-based Docosahexaenoic- and vitamin E-enriched beverage dietary supplementation on inflammation associated to exercise and age. Nutrients.

[52] Calabrese V (2017). Hormesis, cellular stress response and neuroinflammation in schizophrenia: early onset versus late onset state. J Neurosci Res.

